# The Role of Testosterone in the Elderly: What Do We Know?

**DOI:** 10.3390/ijms23073535

**Published:** 2022-03-24

**Authors:** Biagio Barone, Luigi Napolitano, Marco Abate, Luigi Cirillo, Pasquale Reccia, Francesco Passaro, Carmine Turco, Simone Morra, Francesco Mastrangelo, Antonio Scarpato, Ugo Amicuzi, Vincenzo Morgera, Lorenzo Romano, Francesco Paolo Calace, Savio Domenico Pandolfo, Luigi De Luca, Achille Aveta, Enrico Sicignano, Massimiliano Trivellato, Gianluca Spena, Carlo D’Alterio, Giovanni Maria Fusco, Raffaele Vitale, Davide Arcaniolo, Felice Crocetto

**Affiliations:** 1 Department of Neurosciences, Science of Reproduction and Odontostomatology, University of Naples Federico II, 80131 Naples, Italy; biagio193@gmail.com (B.B.); marcoabate5@gmail.com (M.A.); cirilloluigi22@gmail.com (L.C.); reccia.pasquale1@gmail.com (P.R.); francescopassaro1996@gmail.com (F.P.); car.turco87@gmail.com (C.T.); simonemorra@outlook.com (S.M.); f.mastrangelo91@gmail.com (F.M.); antonioscarpato1992@gmail.com (A.S.); u.amicuzi@gmail.com (U.A.); vincemorgera87@gmail.com (V.M.); loryromano@hotmail.it (L.R.); fra.calace@outlook.it (F.P.C.); pandolfosavio@gmail.com (S.D.P.); luigideluca86@gmail.com (L.D.L.); achille-aveta@hotmail.it (A.A.); enrisici90@gmail.com (E.S.); massimiliano.trivellato@gmail.com (M.T.); spena.dr@gmail.com (G.S.); cdalterio95@gmail.com (C.D.); giom.fusco@gmail.com (G.M.F.); felice.crocetto@unina.it (F.C.); 2 Division of Urology, AORN “San Giuseppe Moscati”, 83100 Avellino, Italy; r.vitale0210@gmail.com; 3 Urology Unit, Department of Woman, Child and General and Specialized Surgery, University of Campania ‘Luigi Vanvitelli’, 80131 Naples, Italy; davide.arcaniolo@gmail.com

**Keywords:** testosterone, aging, replacement therapy, erectile dysfunction, metabolic syndrome, depression, hypogonadism, bone density

## Abstract

Testosterone is the most important hormone in male health. Aging is characterized by testosterone deficiency due to decreasing testosterone levels associated with low testicular production, genetic factors, adiposity, and illness. Low testosterone levels in men are associated with sexual dysfunction (low sexual desire, erectile dysfunction), reduced skeletal muscle mass and strength, decreased bone mineral density, increased cardiovascular risk and alterations of the glycometabolic profile. Testosterone replacement therapy (TRT) shows several therapeutic effects while maintaining a good safety profile in hypogonadal men. TRT restores normal levels of serum testosterone in men, increasing libido and energy level and producing beneficial effects on bone density, strength and muscle as well as yielding cardioprotective effects. Nevertheless, TRT could be contraindicated in men with untreated prostate cancer, although poor findings are reported in the literature. In addition, different potential side effects, such as polycythemia, cardiac events and obstructive sleep apnea, should be monitored. The aim of our review is to provide an updated background regarding the pros and cons of TRT, evaluating its role and its clinical applicability in different domains.

## 1. Introduction

**Figure 1:**
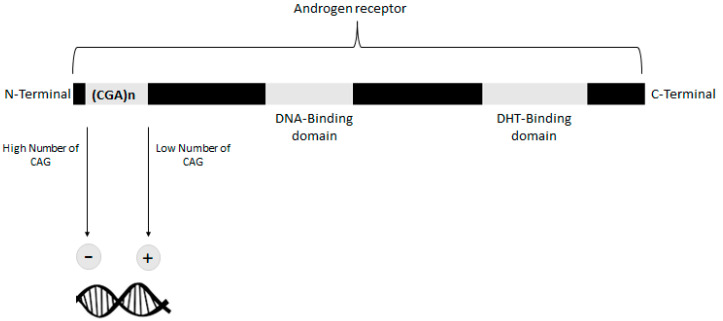
DNA transcriptional activity is influenced by localization of CAG. An opposite association between CAG repeat length and DNA transcriptional activity has been reported. CAG may cause structural perturbations or recruit inhibition proteins.

The passage from adulthood to advanced age represents for men a pivotal point, producing a multitude of physiological, psychological and social changes. One of the most important, and often silent, modifications is related to the change in testosterone values, which could drastically decrease with increasing age. In particular, 25–30% of men > 60 years show low levels of serum testosterone (defined as total testosterone < 350 ng/dL and free testosterone < 225 pmol/L) [1]. Testosterone deficiency is very common in elderly men and is linked to different signs and symptoms (such as reduced libido, reduced sexual functioning, decreases in mobility and energy) that could heavily affect the aging process and the quality of life [2,3,4]. Testosterone effects are mediated via the androgen receptor (AR). Depending on its sensitivity, a significant amount of variations of endogenous and exogenous androgens effects are reported [5]. In particular, CAG repeat polymorphism of AR gene could influence the transcriptional activity of testosterone target genes, consequently providing a multitude of effects on bone density, body composition, behavioral aspects and even prostate cancer [6,7] (Figure 1). 

As result, the use of testosterone replacement therapy (TRT) in clinical practice has been an appealing perspective. However, the issue related to the potential adverse effects of TRT, and in particular its impact on cardiovascular events and mortality, have represented a controversial topic of discussion. Despite those concerns, recent studies have reported non-consistent results regarding the relationship between TRT and increased risk of diabetes, obesity and metabolic syndrome. Contrarily, an adequate replacement therapy appears to improve sexual and physical function, increase bone density and, most importantly, not influence the risk of prostate cancer [8]. In addition, different studies have shown how low endogenous testosterone levels may be associated with reduced cognitive ability and how TRT could help in avoiding cognitive dysfunction associated with hypogonadism [9,10]. Considering the potential benefits of TRT, and, bearing in mind as well, the wide variability of testosterone values and comorbidities in the elderly, TRT should be individualized. The aim of our review is to provide an updated background regarding the pros and cons of TRT, evaluating its role in different domains and its clinical applicability.

## 2. Clinical Findings

### 2.1. Late-Onset Hypogonadism: The Aging Male

Testosterone production and testicular function tend to decrease in aging men by 1–2% per year after the fifth decade [11,12]. Similarly, testicular volume decreases about 15% from 25 to 80–90 years, impacting on quantity and quality of sperm [13,14,15]. Low testosterone levels are indeed associated with decreased sperm production and male sexual dysfunction. In addition, according to the literature, approximately 15% of subfertile men show low serum testosterone and a decreased semen volume and motility compared to eugonadal men [16,17]. Sperm cells morphology could be also negatively affected, with a halved possibility to report a normal morphology (>4% strict Kruger) in men with testosterone serum levels <264 ng/dL [18]. As result, aging men could present mild hypogonadism symptoms, consequently to this relative testosterone deficiency. This condition, which is similar to the hypogonadism of younger men, is characterized by erectile dysfunction, reduced muscle strength, obesity, osteoporosis, hot flushes, fatigue, depression, and poor concentration. The combination of those symptoms, associated with low testosterone levels, has been named in various ways: male menopause, partial androgen deficiency of the aging (PADAM), or late-onset hypogonadism (LOH) (Figure 1) [19].

LOH is a physiological consequence of the aging process, related to the deterioration of the hypothalamic-pituitary-testes axis. The amount and the activity of Leydig cells, which represent the main producers of testosterone, decrease with the age, due to atherosclerosis and degenerative changes [20]. A further blow to Leydig cell functionality could be delivered by the decreasing responsivity to luteinizing hormone (LH), the increased accumulations of free radical-induced damage to components of signal transduction and the age-dependent increases in cyclooxygenase-2 (COX2), which could impair steroidogenesis and redox activity of Leydig cells [21]. COX2, in particular, inhibits the expression of the steroidogenic acute regulatory protein (StAR), a key enzyme in testosterone synthesis [22]. In addition, those phenomena could be further accelerated and influenced by the co-occurrence of different morbidities, such as diabetes, cardiovascular disease and obesity [23,24]. As previously reported, the diagnosis of LOH is based on signs and symptoms of hypogonadism associated with biochemical evidence of low testosterone serum values [25,26]. Notably, although signs and symptoms are mostly related to sexual function, they could also include mild and specific symptoms, such as increased fatigue, irritability, anemia and sleep disturbances [27,28]. As result, to improve the clinical evaluation in the detection of LOH, different questionnaires, embracing other domains in addition to the sexual activity, have been developed, although their low specificity represents a limitation for clinical applicability [29].

### 2.2. Testosterone and Body Composition—Fat and Lean Mass

Among the European population, more than 40% and 25% of obese individuals (BMI, i.e., body mass index, ≥30 kg/m^2^) have total testosterone levels below 345 ng/dL and 300 ng/dL, respectively [30]. In addition, the adipose tissue presents different enzymes, such as aromatase and aldo-keto reductase, that are responsible for metabolizing testosterone into estrogen and 5-dihydrotestosterone, a female hormone and an inactive metabolite, respectively [31]. Testosterone, which is universally recognized as the principal androgen hormone, is characterized by several anabolic and catabolic effects. As an anabolic hormone, testosterone is involved in decreasing visceral adiposity (via the upregulation of AR, which activates the lipolysis) and increasing lean body mass (through the stimulation of Wnt signaling, which promotes differentiation of resident mesenchymal cells into myocytes) [31,32]. Furthermore, testosterone suppresses the differentiation of mesenchymal cells towards the adipose lineage through the inhibition of peroxisome proliferator-activated receptor-gamma (PPARγ) and CAT/enhancer-binding protein alpha (CEBPα), which regulate adipogenesis [33]. Testosterone levels are inversely associated with lean muscle loss and visceral fat redistribution in older men aged ≥65 years and in individuals with obesity [34,35]. Different studies in men receiving TRT reported important effects on the reduction of BMI, waist circumference and bodyweight, as well as on glycometabolic profile [36]. As reported by Saad et al., normalizing serum testosterone in hypogonadal men (administering IM testosterone undecanoate 1000 mg every 12 weeks) delivered a significant decrease in body weight (from 106.22 ± 16.93 kg to 90.07 ± 9.51 kg), as well as waist circumference (from 107.24 ± 9.14 cm to 98.46 ± 7.39 cm) [34]. Similarly, Haider et al. showed, in two independent cohorts of hypogonadal obese men observed for up to 6 years and treated with 1000 mg of testosterone undecanoate IM injections every 12 weeks, a significant decrease in waist circumference, (which decreased by 11.56 cm) and weight (which declined of 17.49 kg) [37]. Nevertheless, there is a lack of randomized clinical trials (RCTs) having TRT-induced weight loss as the primary endpoint, and in the few reported it is labeled as a secondary outcome which is often conflicting or non-significant [38,39]. Results from a previous meta-analysis on the effects of TRT on body composition and other metabolic parameters in LOH are outdated and conflicting [40]. However, a recent meta-analysis by Corona et al., which included 59 RCTs for a total of 3029 patients assuming TRT, showed a significant reduction of fat and fasting glycemia, while lean-mass increased [41]. The results of this study could explain the conflicting findings regarding body weight. Indeed, it is understandable that a reduction in body fat associated with an increase of lean mass could nullify the effects on body weight [42]. The overall body composition would be, however, completely different, as well as the glycometabolic profile [43]. Interestingly, regarding the type of preparation, data showed no improvement in body composition for oral testosterone preparations compared to transdermal and parental preparations. A potential explanation could be related to the different medication adherence which could have further contributed to the differences in effects observed [44].

The role of testosterone on muscle mass has been thoroughly evaluated with its effects which are correlated to testosterone serum levels [45]. The ways testosterone and metabolites affect muscle homeostasis are multiple. First, skeletal muscle converts dehydroepiandrosterone (DHEA) into active androgen hormones and estrogens, which, with the participation of insulin growth factor (IGF-1), regulates muscle growth and repair [46]. Second, testosterone is able to stimulate myocyte activation, proliferation, survival and differentiation [47,48]. Third, testosterone can enhance muscle protein synthesis, leading to an increase in muscle fiber size [49,50]. Finally, testosterone activates, through a G-protein-linked plasma membrane receptor, the Ras/MEK/ERK pathway in muscle cells, leading to the proliferation and growth of myoblasts [51]. In addition, the increase of hemoglobin (of a mean 0.8 g/dL) and myoglobin levels, could further increase muscle function, improving oxygenation [52,53]. Muscle mass begins to decrease 1–2% per year, starting from the third decade, while muscle strength decreases 1.5–3% per year, as a result of a concomitant decline in muscle quality [54,55,56]. The decline of dehydroepiandrosterone sulfate (DHEAS) and testosterone has been found, indeed, to be associated with sarcopenia in the elderly, while the TRT in men with low serum levels of testosterone safely increased muscle mass [57,58]. In addition, TRT was associated with improved muscle strength and power, although it did not influence muscle fatigability or physical function [59]. A study by Page et al. reported, in men >65 years in TRT for 36 months (200 mg of testosterone enanthate every 2 weeks), a significant increase in handgrip strength (3.77 ± 0.55 kg versus −0.21 ± 0.55 kg) and timed functional test (4.3 ± 1.6% versus −5.6 ± 1.9%), compared to placebo [60]. Similar results were obtained for patients >70 years with reduced ejection fraction, when 1000 mg of testosterone undecanoate were administered via intramuscular injection every 6 weeks for 3 months, reporting a peak workload of quadriceps of 88.2 ± 18.7 W compared to 74.3 ± 18 W of the placebo group. Additionally, an inverse linear correlation between testosterone serum levels and oxygen consumption was reported [61]. TRT is beneficial also in intermediate-frail and frail elderly, due to the preservation of muscle thickness, as reported in a study by Atkinson et al., which prescribed transdermal testosterone gel (50 mg per day) to 30 patients >65 years for 6 months, reporting no changes in the treatment group compared to a 1.4 fold decreased muscle thickness of placebo group [62]. Interestingly, beneficial effects of TRT on increased muscle strength and lean body mass were reached also with monthly cycled replacement [63]. To further confirm those findings, a meta-analysis regrouping the effects of 11 TRT RCT studies on lean mass increase reported heterogeneous results, ranging from 1.65-fold to 6.20-fold in patients treated [64]. Finally, although TRT effects on muscle mass and strength were notable for transdermal testosterone formulations, intramuscular TRT is more effective in increasing both variables in middle-aged and older men, particularly in the lower extremities [65,66].

### 2.3. Testosterone and Bone Density

Although the precise mechanism related to androgens and bone density is still unclear, testosterone plays, due to its anabolic effects, a fundamental role in bone growth and maintenance for both women and men [67]. Testosterone increases the proliferation of osteoblasts and, contextually, reduces pro-apoptotic signaling, through a fine regulation of the protein kinase B, while inhibiting osteoclast formation stimulated by parathyroid hormone [68,69]. Age-related testosterone deficiency is associated with a decrease in bone mineral density (BMD) and an increased risk of fracture [70,71,72]. Considering that osteoporosis represents a significant problem in older men and that 30% of all hip fractures occurring in men report increased mortality compared to women, the possibility to maintain testosterone levels within the normal range via TRT is a tempting perspective [73]. Due to these premises, several studies have investigated the beneficial effects of TRT on BMD. Snyder et al. reported, in 108 men over 65 years, an overall increase of lumbar spine bone density in patients treated, for 1 year, with testosterone patches (6 mg of testosterone per day) compared to placebo (4.2 ± 0.8% versus 2.5 ± 0.6%), with a more marked improvement in those reporting low pretreatment serum testosterone values [74]. A similar study by Basurto et al., involving 48 men over 60 years with decreased testosterone levels, reported in the group treated with intramuscular injections of testosterone enanthate (every 3 weeks for 12 months) a significant increment in lumbar BMD compared to the control group (1.198 ± 0.153 to 1.240 ± 0.141 g/cm^2^) [75]. Additionally, TRT could improve trabecular architecture, as assessed at MRI in a small explorative study on 10 hypogonadal men [76]. The efficacy of TRT was also reported for oral testosterone therapy, as reported by Bouloux et al., which investigated the efficacy of 1 year of oral testosterone undecanoate therapy (from 80 mg to 240 mg per day) in 322 aging men in improving BMD at the lumbar spine, hip, trochanter and intertrochanter [77]. Another prospective 2-year randomized trial was performed to evaluate the treatment with oral low-dose testosterone undecanoate (20 mg per day) on BMD of 186 elderly male patients, reporting no significant differences in improving BDM at the lumbar spine and at the femoral neck compared to the full dose (40 mg per day). Interestingly, the high dose group reported the earliest and most homogeneous increase of BMD in the lumbar spine, hip, trochanteric and intertrochanteric sections compared to the low dose which reported an increase only in the trochanter and intertrochanteric sections [78]. The efficacy and tolerability of oral TRT in improving BMD were further demonstrated in an 8-year retrospective study [79]. More recently, another study by Snyder et al. compared the efficacy of transdermal testosterone gel (5 g per day) with placebo, in 211 patients with low testosterone serum values and over 65 years for 12 months. The treatment group showed an increased mean lumbar spine BMD by 7.5% compared to 0.8% of placebo, while the estimated strength of spine trabecular bone improved by 10.8% compared to 2.4% [80]. However, despite the findings of reported studies, the efficacy of TRT in improving BMD and preventing fracture remains controversial. As reported by a recent meta-analysis on 52 RCTs by Zhang et al., testosterone supplementation did not increase total BMD when compared to placebo, although a high heterogeneity in treatment modality and duration < 2 years was reported. Notably, patients involved did not report osteoporosis and it is well known that the effects of TRT could be evident after 2–3 years [81]. Further studies with longer follow up are required to properly evaluate the role of TRT on bone density.

### 2.4. Testosterone and Sexual Activity

Testosterone is involved in every step of male sexual response, from sexual desire to erectile function, although sexual disorders cannot be automatically related to its decline during aging [82,83,84]. In addition, low testosterone levels could further indirectly impact sexual activity, producing decreased energy, depressive symptoms and fatigue [85]. Erectile dysfunction (ED) is one of the most important symptoms related to aging and low testosterone levels. However, despite erections being clearly androgen-dependent, the level of hypogonadism required to induce ED is debatable and could be influenced by external factors [86,87]. Nevertheless, TRT seems to positively influence erectile function, as reported in a recent meta-analysis by Corona et al. which showed, in 14 clinical trials (for a total of 2298 patients involved), a significant increase of International Index of Erectile Function-Erectile Function Domain (IIEF-EFD) score of 2.31 (95% C.I 1.41–3.22) in patients treated with TRT compared to placebo. This increase was even higher in patients who reported mild and severe testosterone deficiency [88]. As reported by Saad et al. in a cohort of 805 hypogonadal men, the effects of TRT (1000 mg of testosterone undecanoate IM every 12 weeks), on erectile function are more and more sustained up to 9 years of replacement therapy, in addition to significant improvement in cardiometabolic risk factors while Efesoy et al. suggested the use of TRT in restoring penile hemodynamics in hypogonadal men with veno-occlusive dysfunction [89,90]. The use of TRT has also been investigated in association with phosphodiesterase type 5 inhibitors (PDE-5i), reporting a significant advantage, in terms of IIEF-5, of combined therapy [91,92]. As reported in a recent meta-analysis by Zhu et al., comprehending eight studies for a total of 913 hypogonadal patients involved, the combination therapy delivered an overall higher standard mean deviation for erectile function component change (0.663, 95% CI 0.299–1.027) compared to PDE-5i alone. Interestingly, the frequency of adverse effects was comparable in both groups and none of the patients reported an increase in prostate-specific antigen above 4 ng/mL [93].

In addition to the erectile function, TRT could also influence ejaculatory function. Orgasmic and ejaculatory dysfunctions constitute, indeed, a spectrum of ejaculatory symptoms which comprehend retrograde ejaculation, reduction in ejaculate volume and decreased force of ejaculation. Although several comorbidities could affect ejaculatory function, low serum testosterone levels contribute to its alteration [94]. A recent double-blinded, randomized and placebo-controlled trial, involving 76 men with one or more ejaculatory dysfunction symptoms, reported a positive effect (improving IIEF of a mean 3.1 points), although not statistically significant, of TRT (60 mg of 2% testosterone gel per day) on those alterations [95]. Testosterone could, indeed, plays a facilitatory role in the control of ejaculatory reflex, acting on a central and peripheral mechanism [96].

Regarding the influence of TRT on reduced libido, Cunningham et al. conducted a placebo-controlled trial on a total of 470 men with low libido over 65 years of age, reporting, in patients who underwent TRT (5 g of 1% testosterone gel per day) a significant improvement in 10 of 12 measures of sexual activity (assessed via three questionnaires—the Psychosexual daily questionnaire, the Derogatis interview for sexual function and the International Index of Erectile Function) [97]. A similar study involving 715 males with total testosterone lower than 300 ng/dL and at least one symptom of testosterone deficiency, reported, in patients treated with topical testosterone, a higher increase in Sexual Arousal, Interest and Drive Scale (SAID) and Hypogonadism Energy Diary (HED) compared to placebo [98]. Brock et al. also evaluated the safety and efficacy of topical testosterone 2% gel daily on 558 hypogonadal men, showing, after 6 months, a significant increase of SAID and HED scores [99]. Interestingly, none of these trials found a significant threshold below which libido was universally affected for men in the study.

### 2.5. Testosterone and Glycometabolic Profile—The Effects on Insulin Sensitivity

Although testosterone levels and insulin sensitivity decrease with age, little is known about this interaction in men. Several cross-sectional studies demonstrated an inverse correlation between testosterone and insulin levels, outlining a positive relationship between testosterone and insulin sensitivity [100,101]. More recently, a large systematic review and meta-analysis showed that testosterone level is significantly lower in men with T2DM (mean difference, −76.6 ng/dL; 95% CI, −99.4, −53.6) [102]. To further support those findings, Atlantis et al. reported, in a prospective study, that low serum testosterone predicts an increased risk of developing T2DM over 5 years [103]. Furtherly, Rubinow et al. showed how acute testosterone deprivation reduced insulin sensitivity in men [104]. As result, a growing interest in the potential use of TRT in patients with T2DM has risen. Unfortunately, a study by Magnussen et al. reported no effects on peripheral insulin sensitivity nor endogenous glucose production of TRT in aging men with lowered testosterone levels [103]. Similar results reported a recent study by Huang et al. on 308 community-dwelling men over 60 years with low or low-normal testosterone levels [105]. The randomized, double-blind, case-control study T4DM examined patients with impaired glucose tolerance or recently diagnosed T2DM, reporting a reduction of patients with T2DM in those in treatment with testosterone for 2 years, in addition to an adequate lifestyle program. In particular, the mean change from baseline 2-h glucose was −0.95 mmol/L in the placebo group and −1.70 mmol/L in the testosterone group, with a mean difference of −0·75 mmol/L [106]. Despite the evidence, the issue is still controversial and needs to be clarified. As reported by an extensive meta-analysis by Corona et al., metabolic syndrome patients showed significantly lower testosterone levels compared to healthy individuals and both T2DM and metabolic syndrome independently predicted low testosterone (r = −0.752 and −0.271, respectively) [107]. Conversely, TRT seems to reduce HbA1c (MD = −0.67, 95% CI −1.35, −0.19) and improve homeostatic model assessment of insulin resistance (HOMA-IR) (standard mean deviation = −1.94, 95% CI −2.65, −1.23) [108].

The mechanisms mediating the effects of testosterone are multifold and still uncleared. In addition, various mechanisms could contribute to increased insulin sensitivity after testosterone therapy, such as loss of subcutaneous fat, gains in muscle mass and suppression of inflammation. Blava et al. showed, for example, in a cross-sectional study with 143 non-diabetic men over 60 years, a strong association between low total testosterone and visceral adiposity. The results showed that 47.9% of the subjects had metabolic syndrome, and the proportion of subjects with metabolic syndrome was higher among those with low total testosterone [109]. Angelova et al., instead, focused on the connection between total testosterone levels, metabolic syndrome and IL-18, in a population of 218 men with metabolic syndrome with 33 healthy controls. Most of the subjects with metabolic syndrome had type 2 diabetes (87%), hypertension (81.6%), and lipid disorders (75%), with a low total testosterone found in 38.5% of patients. Subjects with metabolic syndrome had higher IL-18 levels compared to the healthy control group, but no significant difference in IL-18 levels was found between subjects with metabolic syndrome and low or normal testosterone serum levels [110]. Due to the simultaneity of those changes, it is not possible to determine the relative contributions of either mechanism on overall insulin sensitivity [111].

### 2.6. Testosterone and Cardiovascular Disease

Testosterone has an important role in cardiovascular homeostasis, with a cardio-protective effect via its activity on lean body mass, body composition, changes in lipid profile and insulin resistance [112]. Several epidemiological and observational studies have shown a direct association between low serum endogenous testosterone and increased risk of cardiovascular disease [113,114]. In addition, a large meta-analysis including 70 studies, indicated that patients with cardiovascular disease demonstrated significantly lower T and higher 17-b estradiol levels [115]. Another extreme example of acute hypogonadism in men is patients receiving androgen deprivation therapy (ADT) for prostate cancer. In this regard, a joint scientific statement from the American Heart Association, American Cancer Society and American Urological Association published in 2010, suggested a possible association between ADT and risk of CV events [116]. Nevertheless, there are no currently clear associations between the effects of TRT and cardiovascular risk. Indeed, results from recent meta-analyses have been widely contradictory, ranging from the increased cardiovascular risk associated with TRT to protective effects of TRT in patients with metabolic syndrome and other cardiovascular risk factors [117,118]. The main cohorts in which cardiovascular risk associated with TRT could be investigated are those assuming TRT for hypogonadism issues. Although there seem to be no major complications in using TRT in young and healthy men, its use in older men and in patients with known cardiovascular disease remains unclear, advising reasonable caution in proposing TRT in those cases [119]. Similarly, controversial results are reported regarding the effects of TRT on cardiovascular events and mortality. A comprehensive review of the literature reported, however, an overall neutral effect of TRT on the occurrence of major cardiovascular events [120].

The study of Basaria et al. was one of the first clinical trials which underlined potential cardiovascular issues related to TRT. The trial, designed as a randomized, placebo-controlled, involved 209 men over 65 years and was designed to evaluate the effects of TRT administered via testosterone gel, for 6 months. During the course of the study, the testosterone group had a higher rate of cardiac, respiratory and dermatologic events compared to the placebo group [121]. An analogous study was affected by Finkle et al. which reported, in 55,953 patients who received TRT, an increased risk of developing myocardial infarction. In particular, the rate of non-fatal myocardial infarction within 90 days of TRT, was higher in men over 65 years (OR 2.19; 95% CI: 1.27–3.77) and further increased when patients over 75 years were considered (OR 3.43; 95% CI: 1.54 to 7.56) [122]. Conversely, Baillargeon et al. did not describe an increased risk of myocardial infarction in a cohort of men > 65 years assuming TRT via intramuscular injections compared to placebo while Sharma et al., which investigated the effects of TRT on 83,010 hypogonadal male veterans, reported a significant decrease of all-cause mortality (OR 0.44; 95% CI: 0.42–0.46), risk of myocardial infarction (OR 0.76; 95% CI: 0.63–0.93) and stroke (OR 0.70; 95% CI: 0.51–0.96) in those receiving TRT [123,124].

Many reasons underlie the controversial results regarding cardiovascular risk and TRT, including differences in inclusion and exclusion criteria, baseline testosterone serum values and degree of clinical hypogonadism. In addition, the differences in studies regarding the duration of follow-up, variability in the definition of major adverse cardiovascular events as well as the type of TRT preparations, could further influence the uncertainty of data reported in the literature.

On 17 September 2014, the U.S. FDA convened an advisory committee to review the use of TRT and its cardiovascular risks. The committee was asked to give an opinion on the current indications for TRT and whether manufacturers of testosterone products should conduct studies to assess cardiovascular risks associated with the use of TRT. The committee concluded that there was not enough evidence that TRT was a significant CV risk for any given group of patients treated with TRT. The committee commented, however, that TRT safety in high CV risk groups, such as elderly, diabetic, and obese men, needed further studies. In March 2015, the FDA further clarified recommendations for the use of TRT, stating its approved use only in men with documented low testosterone levels caused by specific medical conditions, while its use in elderly with low testosterone levels, even if symptomatic is still unclear [125].

### 2.7. Testosterone and Mental Activity

The role of sex hormones in influencing mental activity and mood is well established [126,127]. Testosterone seems to support psychological features related to good mood and enjoyable quality of life [128]. Indeed, different studies reported how the rate of dysphoria, irritability, lack of assertiveness and depression is higher, compared to healthy controls, in older men with low testosterone serum values [129,130]. The rationale resides in the neuroactive activity of testosterone which influences mood, appetite and serotonin release, in addition to being involved in neuroplasticity processes of the hippocampal formation [131,132]. Interestingly, the role of testosterone is not limited to the dichotomous negative-positive mood but influences other several and complex behaviors, such as unconscious fear and anxiety, social dominance (via aggressive and not behaviors) and even parochial altruism, i.e., intergroup cooperation and outgroup hostility [133,134,135].

The association between depressive symptoms and aging males is a controversial topic. Although the decreased serum levels of testosterone could impact the overall mood, it is also true that many comorbidities could represent an important bias on the role of testosterone in depression [131]. However, considering that the elderly who are depressed appear to have low testosterone levels compared to eugonadal men, the possible role of TRT in mental health has been postulated [136]. Several studies show, indeed, an association of testosterone reduction in older men (which could be functional or late-onset hypogonadism related) and depressive symptoms ranging from dysthymia, fatigue and inertia to listlessness, hopelessness and suicidal thoughts [128,137]. In fact, a greater prevalence of these symptoms can be observed in elderly individuals, an age group in which hormone levels reach the lowest levels and occur with andropause [138]. As reported by Giltay et al., in a randomized, placebo-controlled, double-blind trial in 184 hypogonadal men receiving intramuscular testosterone undecanoate versus placebo, demonstrated indeed a marked decrease in the Beck Depression Inventory in men receiving TRT (mean difference vs. placebo after 30 weeks: −2.5 points; 95% CI: −0.9; −4.1) [139]. Similarly, Snyder et al., in a larger double-blind placebo-controlled trial involving 788 functional hypogonadal men > 65 years, reported an improvement in depressive symptoms, as demonstrated by lowered scores on the Patient Health Questionnaire 9. In particular, the treatment delivered a decrease of −0.72 (95% CI: −1.20 to −0.23) compared to the placebo [140]. A recent meta-analysis comprehending 27 randomized controlled trials for a total of 1890 men involved, demonstrated that TRT is associated with an important reduction in depressive symptoms compared with placebo showing an odds ratio of 2.30 (95% CI: 1.30–4.06) [141]. Similar findings were also found in a previous meta-analysis which, in addition, showed greater efficacy of TRT in subpopulations with hypogonadism, HIV and patients treated with transdermal formulations [142]. Furthermore, TRT could be used as a second-line treatment for SSRI-refractory major depression in men [143]. Despite those findings, the real effect of TRT is still difficult to measure and its clinical implications are not fully clear [144].

The associations of anxiety and panic disorders with testosterone have not been fully investigated. Nevertheless, hypogonadal men exhibit a significantly higher prevalence of anxiety disorders while the administration of a single dose of testosterone showed an anxiolytic effect in females [142,145]. An inverse correlation between anxiety symptoms and free testosterone was reported, in a cohort of 3413 men, by Berglund et al., which showed a higher symptom score in testosterone deficient men compared to eugonadal men [146]. Conversely, in a smaller study involving 296 older men by Schneider et al., testosterone has been shown to increase the risk of panic and phobic anxiety in patients with a genetically determined long CAG repeat polymorphism of the androgen receptor [137]. Regarding the effects of androgens in the elderly, it has been reported that low testosterone serum levels are independently associated with incident dementia and Alzheimer’s disease, confirmed by an increased incidence of those conditions in ADT patients [147,148]. Conversely, further evidence is required to enlighten the relation between testosterone and Parkinson’s disease, considering the neuroprotective effects of 5-alfa reductase inhibitors in animal models [149].

Regarding the relationship between testosterone and aggressiveness, TRT did not increase self- and partner-reported aggression. The use of testosterone supplementations and their effects on aggressivity is well known from misuse in sports. At the beginning of the new millennium, Pope et al. demonstrated, in an interesting RCT, how a short course of 600 mg/week of testosterone cypionate for 6 weeks, significantly increased manic and aggressive response in eugonadal men between 20–50 years [150]. A recent meta-analysis by Chegeni et al., comprehending 12 RCTs for a total of 562 healthy males, confirmed aggressivity and irritability as important psychiatric side effects of anabolic-androgenic steroid use [151]. Despite those findings, the link between testosterone and aggression, violence and even antisocial personality, is complex and remains controversial, although different hypotheses have been formulated related to withdrawal effects and supraphysiological doses of testosterone [152]. Interestingly, as reported by O’Connor et al., the administration of 200 mg intramuscular testosterone enanthate weekly for 8 weeks in hypogonadal men, compared to placebo, did not increase aggression and impulsivity. Conversely, negative mood, anger and fatigue were significantly reduced [153]. A similar pilot study by Kenny et al. on 11 elderly men (>70), treated with 200 mg intramuscular testosterone enanthate every 3 weeks for 12 weeks, showed no increase in aggressiveness or unwanted behaviors [154]. More recently, in a meta-analytic examination of baseline, dynamic and manipulated testosterone on human aggression, Geniole et al. reported no evidence of effects of exogenous testosterone on human aggression, although a high heterogeneity of studies was underlined [155].

### 2.8. Testosterone and Quality of Life

Quality of life (QoL) is a set of psychological variables that together contribute to the individual perception of a state of complete physical, mental, and social well-being, each including both cognitive and emotional components [156]. Testosterone plays an important role in the maintenance of QoL, supporting psychological features, behavior, self-perception and perceived quality of life [3]. A further example is related to sleep disturbances, which represent an important and influencing factor in QoL, and are inversely related to serum testosterone levels, which are lower in patients with worsened quality of sleep and higher frequency of nocturnal awakenings [157]. According to recent literature, TRT significantly improved quality of life (standardized mean difference −0.26, 95% CI: −0.41; −0.11) compared to placebo. although effects were a bit attenuated in men with symptoms of depression [158]. In a recent meta-analysis including sixteen trials for a total of 944 subjects, an overall positive impact of TRT was reported on general mood, with an age-dependent effect size of z = 4.592. No differences were reported regarding the types of TRT preparations [159]. A double-blind, placebo-controlled clinical trial performed by Behre et al., involving 362 hypogonadal men ranging from 40 to 79 years assuming TRT via transdermal testosterone gel, reported marked changes compared with placebo in health related QoL, in addition to the effects on the physical composition, within 6 months [160]. A similar trial, performed by Tong et al., confirmed the effects of TRT, without increasing potential adverse effects, also in the short-form-12 (SF12) scale analysis of QoL, with an overall increase of 4.4 points from the baseline in patients treated compared to placebo [161]. Finally, in a large multinational study, involving 999 untreated men with hypogonadism, treated with TRT, a significant, rapid and sustained improvement in QoL, assessed via the Aging Males’ Symptom (AMS) scale, was reported, up to 36 months after the initiation of treatment [162].

Despite that evidence, the role of testosterone in influencing QoL remains very controversial and limited, due to the wide variability of assessments and patient baseline characteristics. More studies and larger samples are required, in order to properly evaluate the effects of TRT on QoL, permitting to balance the risks and the benefits of the therapy.

### 2.9. Testosterone and Prostate Cancer 

Testosterone is an essential hormone for the normal growth of the prostate. Even if available since the early 1940s, testosterone therapy has always met resistance due to the belief that it may promote prostate cancer growth, due to the association between increased serum marker acid phosphatase and testosterone and the rapid decrease of this marker following castration of men with metastatic prostate cancer [163]. However, according to this belief, high testosterone concentrations would be associated with an increased risk of prostate cancer while low testosterone concentrations should have a protective effect. Nevertheless, hypogonadal men did not have lower cancer rates compared to eugonadal populations, additionally, testosterone levels are not elevated in patients with prostate cancer [164]. This apparent paradox was resolved by Morgentaler et al. who highlighted that the concentration of testosterone in men with prostate cancer was based on the concept of prostate cancer tumoral cells sensitivity, regardless of testosterone concentration. This concept is summarized as the saturation model, which accounts for the key observation that prostate cancer growth is sensitive to variations in serum testosterone levels at or below the near-castrate range and is insensitive to testosterone variation above this threshold [165]. To further complicate the role of testosterone, in situ production of estrogens due to aberrant expression of aromatase in prostate cancer (added to the aromatase already expressed in the adipose tissue), could modify the ratio between circulating and intraprostatic estradiol/testosterone [166]. Finally, it is well known that the effects of testosterone could widely vary depending on the variants of its receptor [167]. If this is important in its biological effects on reproductive, musculoskeletal and hemopoietic systems, it is crucial in prostate cancer, as AR variants influence the efficacy and prognosis of androgen deprivation therapy (ADT) [168,169].

**Figure 2:**
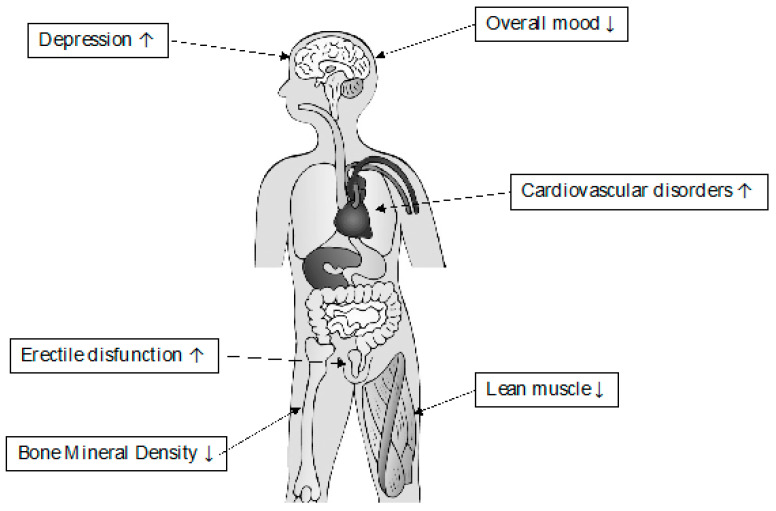
Effects of Testosterone deficiency in the elderly.

As result, in an interesting meta-analysis, Cui et al. analyzed the effect of TRT on prostate cancer in eight trials grouped into men undergoing TRT < 12 months and between 12 and 36 months. Even if a reduced risk of prostate cancer was found in the first group of trials and a null risk of prostate cancer was found in the second group of trials, no statistically significant differences emerged [170]. Regarding the role of TRT in patients with diagnosed prostate cancer, Kaplan et al. analyzed the safety of TRT in 1181 men receiving exogenous testosterone following their cancer diagnosis. Results showed no association between the testosterone treatment after prostate cancer diagnosis with overall mortality, cancer-specific mortality, or the subsequent use of androgen deprivation therapy [171]. Similarly, in a previous study, Agarwal et al. reported, in a cohort of hypogonadal patients treated with radical retropubic prostatectomy (RRP) for organ-confined prostate cancer who presented low serum total testosterone and symptoms of hypogonadism, no PSA recurrence and statistically significant improvements in total testosterone and hypogonadal symptoms after 19 months of TRT [172]. Finally, Lenfant et al. systematically reviewed studies on testosterone administration in men with a known prostate cancer history, underlining that the available evidence does not suggest an increased risk of recurrence or progression of prostate cancer in patients undergoing TRT. However, there is a lack of evidence on the safety of long-term TRT [173]. Lastly, no evidence is reported regarding the interaction, or contrast, between TRT and ADT [174] (Figure 2).

## 3. Safety of TRT

Despite the beneficial effects of TRT being widely reported and publicized in literature, a paucity of findings on TRT side effects are currently reported. If it is true that the effects of TRT on prostate enlargement, prostate cancer and lower urinary tract symptoms are controversial, it has been reported that there is a significant increase in hemoglobin of 5–7%, secondary to testosterone-induced polycythemia [175,176,177]. As a result, it has been supposed that there is an increased risk of vascular events related to this condition, which, however, has not been demonstrated in men on TRT [52,178]. Additionally, although it has been reported an increased risk of obstructive sleep apnea in patients on TRT, its etiology is not fully understood, and findings are inconsistent [179,180]. As result, TRT has considered, overall, a safe therapy, albeit a proper follow-up should be required to check hematocrit [181] (Table 1). 

## 4. Future Perspectives and Conclusions

Testosterone plays a pivotal role in the integrity and maintenance of systems and organs of the aging male. Aging is, indeed, characterized by a wide variety of declines in testosterone production, which could heavily affect health and quality of life (Table 2). The use of TRT in older patients, to normalize testosterone levels, is a tempting perspective that could provide several important benefits in terms of sexual life, mental activity, metabolism, and bone/musculature integrity. Many factors could modulate androgen production and effects in the elderly. However, a lack of reliable and practical markers limits the possibility to assess the real action of exogenous testosterone in tissue and organs. In our opinion, an effort should be directed towards the identification of patients who could obtain more benefits from TRT. This objective could be further and ideally assisted by the identifications of AR polymorphisms. In this regard, different ongoing clinical trials are exploring the role of TRT in the aging male (as NCT02873559, NCT04743466 and NCT04584918). In addition, the development of several testosterone preparations has increased the personalization and compliance of TRT [182]. Future research should, therefore, focus on a tailored and multidisciplinary approach, in order to balance the benefits and risks of TRT.

## Figures and Tables

**Table 1:** Effects of Testosterone Replacement Therapies.

Author	Study Design	Sample Size	Aim of the Study	T Formulation	Outcome
Saad, 2013 [34]	P	255	T effect on anthropometric parameters in hypogonadal men	T undecanoate	Improved BW, WC, and BMI
Haider, 2014 [37]	P	156	T effect in obese men and type 2 diabetes mellitus with T deficiency	T undecanoate	Improved HbA1c, WC, BMI
Snyder, 1999 [74]	RCT	108	T effect on bone density	T gel	Improved Lumbar BMD
Basurto, 2008 [75]	RCT	48	T effect on bone density healthy elderly men with low levels of total testosterone	T enanthate	Improved Lumbar BMD
Saad, 2019 [89]	P	420	T effect on ED in hypogonadal men	T undecanoate	Improved EF
Paduch, 2015 [95]	RCT	76	T effect on ED in hypogonadal men	T gel	Improved EF
Wittert, 2021 [106]	RCT	504	T effect on early type 2 diabetes	T undecanoate	Improved Type 2 diabetes
Sharma, 2015 [124]	R	83 010	T effect on cardiovascular system	T undecanoato/gel	Decreased All-cause mortality, MI, and stroke.
Giltay, 2010 [139]	RCT	184	T effect on depressive symptoms	T undecanoate	Decreased Depression symptoms
Behre, 2015 [160]	RCT	362	T effect on Quality of life in hypogonadal men	T gel	Improved Quality of life
Kaplan, 2014 [171]	O	1181	T effect in men with prostate cancer.	T undecanoate	No improve Cancer-specific mortality

BDM: bone mineral density; BMI body mass index Body weight (BW); EF: erectile function MI:myocardial infarction; O: observational study; P: prospective study; R: Retrospective study; RCT: randomized controlled trial; WC: waist circumference.

**Table 2:** Signs and symptoms related to low testosterone levels.

Signs and Symptoms Related to Low Testosterone Levels	
Bone Density	Osteoporosis Decreased resistance to fractures
Mental Activity	Depression Poor concentration Fear Anxiety
Body Composition	Low muscle mass Decreased strength Increased muscular fatigue
Glycometabolic profile	Obesity Insulin resistance Metabolic Syndrome
Cardiovascular disease	Hot flushes Increased risk of atheroma development Endothelial dysfunction
Sexual Activity	Erectile dysfunction Low desire Ejaculation disorders

## Abbreviations

TRT	Testosterone Replacement Therapy
PADAM	Partial Androgen Deficiency of the Aging Male
LOH	Late-Onset Hypogonadism
LH	Luteinizing hormone
COX2	cyclooxygenase-2
StAR	steroidogenic acute regulatory protein
BMI	Body Mass Index
AR	androgen receptor
PPARγ	Peroxisome Proliferator-Activated Receptor-gamma
CEBPα	CAT/enhancer-binding protein alpha
IM	intramuscular
RCT	Randomized Clinical Trial
DHEA	Dehydroepiandrosterone
DHEAS	Dehydroepiandrosterone sulfate
BMD	bone mineral density
IIEF	International Index of Erectile Function
EFD	Erectile Function Domain
PDE-5i	phosphodiesterase type 5 inhibitors
SAID	Sexual Arousal, Interest and Drive Scale
HED	Hypogonadism Energy Diary
T2DM	Type 2 diabetes mellitus
HbA1c	glycated hemoglobin
HOMA-IR	homeostatic model assessment of insulin resistance
IL-18	interleukin 18
ADT	Androgen deprivation therapy
CV	cardiovascular
FDA	Food and drug administration
QoL	Quality of life
AMS	Aging Males’ Symptom
ED	Erectile dysfunction
